# Acute Effects of Block Jumps in Female Volleyball Players: The Role of Performance Level

**DOI:** 10.3390/sports5020030

**Published:** 2017-05-16

**Authors:** Javier Brazo-Sayavera, Pantelis Theodoros Nikolaidis, Alba Camacho-Cardenosa, Marta Camacho-Cardenosa, Rafael Timón, Pedro R. Olivares

**Affiliations:** 1 Research Group on Advances in Sport Training and Physical Conditioning, University of Extremadura, Cáceres 10003, Spain; albacc@unex.es (A.C.-C.); mcamachocardenosa@unex.es (M.C.-C.); rtimon@unex.es (R.T.); 2 Higher Institute of Physical Education, University of the Republic, Rivera 40000, Uruguay; 3 Exercise Physiology Laboratory, Nikaia 18450, Greece; pademil@hotmail.com; 4 Instituto de Actividad Física y Salud, Universidad Autónoma de Chile, Talca 1670, Chile; polivaress@uautonoma.cl

**Keywords:** plyometric training, stretch-shortening cycle exercise, athletic performance, physical fitness

## Abstract

Although the role of jumping ability in female volleyball players is well recognised, the effect of fatigue on this ability is not well known. The aim of the present study was to examine the effect of a series of block jumps (BJ) on jumping ability and whether it varies by performance level. Ten elite (EG) and 11 amateur (AG) female volleyball players performed a fatigue intervention consisting of 45 BJ, being tested for squat jump (SJ), countermovement jump (CMJ) and BJ before and after the intervention. Elasticity index (EI): (100 × (CMJ − SJ)/SJ) and upper limbs coordination index (ULCI): (100 × (BJ − CMJ)/CMJ) were calculated. After the intervention, EG showed a decrease of 4.40% in BJ height (*p* = 0.04; ES = 0.40), whereas AG presented an increase of 1.27%, which was not significant (*p* = 0.57; ES = 0.07). However, EG and AG presented no significant differences in SJ (*p* = 0.965 and *p* = 0.655) and CMJ (*p* = 0.742 and *p* = 0.211) when comparing baseline with post-intervention. Although EI and ULCI showed no significant differences after intervention in any group (AG: *p* = 0.989 and *p* = 0.114; EG: *p* = 0.242 and *p* = 0.205, respectively), AG presented a medium effect size (ES = 0.50) in EI and a small one in ULCI (ES = 0.37), also EG showed a medium-large effect in ULCI (ES = 0.75). These findings suggest that EG performance in BJ tends to decrease at the end of a specific jump training practice. Therefore, coaches and fitness trainers working with elite volleyball players should focus on exercises to maintain jumping ability during a match.

## 1. Introduction

Vertical jumps (VJ) are frequent actions in volleyball practices and games, playing a very important role in both attack and counterattack [1]. Block and spike jumps are the most important skills in determining team success in volleyball [2]. Spike and block jump (BJ) effectiveness depends on the height at which these jumps are performed above the net [3]. According to previous researchers, these jumps should be tested in volleyball players because they are crucial skills in the game [2,4].

Volleyball players can start block jumps using a countermovement action or using a squat position, although during actual games, the more common type of static block jump utilized by players is the squat BJ [5], even knowing that the countermovement jump (CMJ) achieves a greater height than the squat jump (SJ) [6]. The starting position affects the use of the combination of eccentric and concentric actions, stretch-shortening cycle (SSC), or only concentric, respectively. The elastic and contractile components of muscle play a vital role during the SSC phenomenon. Due to SSC, strain energy is stored in the elastic tissue of muscle during CMJ actions, reaching a higher jump in comparison to SJ actions [7,8,9] if the concentric part of SSC is followed immediately after the eccentric one [10]. However, if the coupling with the velocity of stretch is too short, it is most likely to affect the performance potentiation in SSC. Hence, the nervous system helps to regulate muscle stiffness and muscle elasticity utilization in ballistic exercises [11]. It is therefore important to pay attention to the role of the muscle spindle and Golgi tendon organs as controllers in SSC actions in order to protect the muscle-tendon complex [12]. The reflex produced during the eccentric phase of the SSC contributes significantly to generating of force in subsequent concentric muscle actions [13]. However, it has been suggested that repeated long-term SSC generates neuromuscular fatigue and reducing muscle stiffness affecting neuromuscular function and in consequence affecting natural actions such as jumping [14]. Hence, SSC performance predicts results in the jumps associated with volleyball performance in elite players [15], and muscular fatigue could affect technical actions [16]. There is a link between the level of jump height and fatigue in female volleyball players. Nikolaidis et al. [17] showed that female volleyball players with better jump height in an Abalakov jump had less fatigue index in a Wingate anaerobic test. Therefore, maximal CMJ height has been assessed after reaching the point of volitional fatigue in order to determine a resting interval between CMJs that allows the maintenance of maximal jumping performance. Pereira et al. [18] concluded that an adequate resting period (around 14 s) between spikes allows volleyball players to maintain their performance. Fourteen seconds is the average resting period during a match, not including player substitutions or timeouts, hence players should maintain their performance during a match. [19].

Strength, speed, and power are dominant in spiking and blocking actions, and in most cases, winning points depend on them [20]. It has been shown that changes in physical performance occur after an in-season training regimen in professional female volleyball players [21], but the changes in VJ performance during a game have yet to be investigated. Knowledge about changes of VJ during a game is important for coaches and fitness trainers working with volleyball players. Such knowledge helps them understand the link between fatigue and VJ and help them tailor training programs accordingly. Also, the information about whether this link varies with volleyball players’ performance level is of practical importance [2]. Knowing that female players average 45 jumps during two games, with a 73 jumps maximum [22], the aim of this study was to determine the effects on jump height of a workout based on block jumps on female volleyball players with different competition levels. It was hypothesized that (1) a significant decrease in jump height on different tests (SJ, CMJ and BJ) would be observed after the intervention (especially in amateur players), (2) no change in the elasticity index would be observed, and (3) a decrease in the upper limbs coordination index would be observed.

## 2. Materials and Methods

### 2.1. Subjects

**Figure 1:**
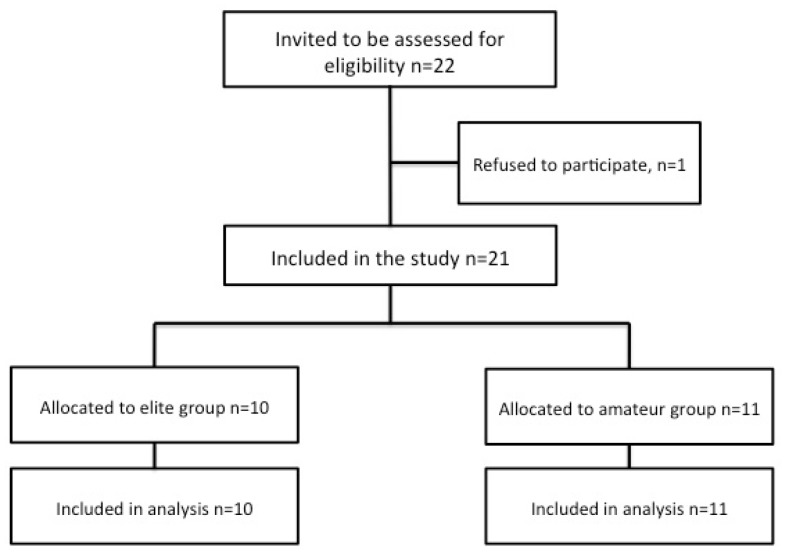
Flow diagram of the study participants.

Two female volleyball teams participated in this study (Figure 1): an elite team (EG, *n* = 10; age: 25.8 ± 4.4 years) and an amateur team (AG, *n* = 11; age: 20.8 ± 3.4 years). EG subjects finished eighth in the Spanish volleyball First League during the 2014–2015 season. AG subjects finished in first place in the National Second Division League (fourth division) during the same season and all of them were amateurs. EG subjects had four training sessions per week, whereas AG subjects had three training sessions per week. The subjects were cleared of any medical disorders that might limit their full participation in the study. This study was carried out during the last week of the competitive period. The subjects and coaches were informed in detail about the experimental procedures and the possible risks and benefits of the project, which was approved by the Bioethics Committee of the University of Extremadura and carried out according to the Declaration of Helsinki.

### 2.2. Data Collection

Subjects conducted familiarization sessions on the jump procedures used in the study three days prior to testing. All subjects within a given team were assessed on the same day and all three different types of jumping performances were carried out in a random order. Testing was integrated into weekly training schedules. Each subject carried out the protocol after the previous one had finished. Before each test, the subjects performed 10 min of warm-up and 5 min of their traditional sport-related dynamic stretching routine. They did not do any maximal vertical jumps in order to eliminate any influences on their subsequent jumping performance.

**Figure 2:**
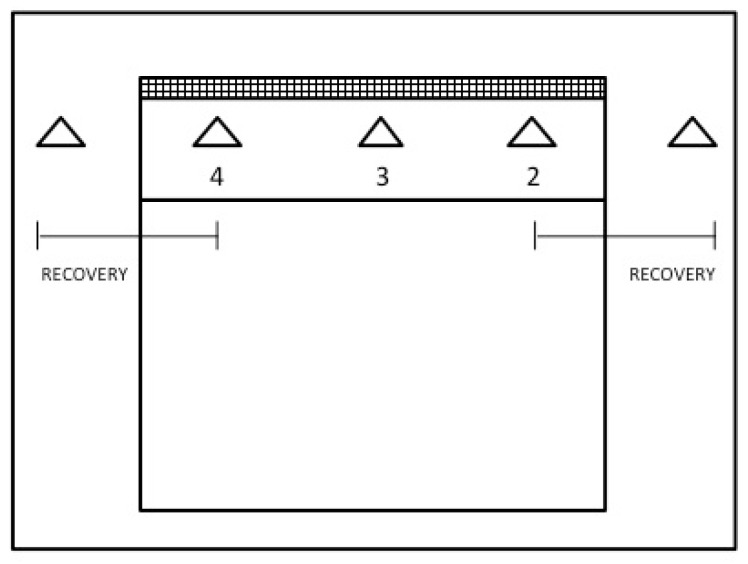
Schematics of the experimental intervention.

After a warm-up of approximately 10 min with varying medleys, a block with technical drill segment followed up. After testing the baseline jumps, subjects were required to complete 45 block jumps divided in 15 bouts of three jumps. Each jump was performed near the net separated by cones (the three attack zones). Subjects moved free between cones after landing to perform next jump. Subjects had a recovery zone of 8 m between bouts (Figure 2). Immediately after the intervention, subjects started the second part of the testing protocol.

The jumping test was performed on an indoor court and consisted of three different maximal jumps on an optical measurement system (Optojump, Microgate, Bolzano, Italy). The subjects were asked to perform a maximal jump on the optical measurement system from the starting position.

To do the squat jump, they started from 90-degree knee flexion. This position was maintained for about 1 s. Then, subjects were instructed to extend the lower limbs, as explosively as possible. To perform the countermovement jump they started from a standing position with a preparatory movement from the extend leg position down to the 90-degree knee flexion followed by a subsequent concentric action. To perform SJ and CMJ, hands were placed on a wooden stick on the shoulders. The BJ started from a standing position with the hands in front of the subject at shoulder level and arms raised from the start position. Subjects raised their arms directly from this position, without an extra swing, to get help to perform the jump. After that, they adopted the squat position (90-degree knee flexion) and they maintained it for about 1 s before jumping. Subjects were instructed to land on the platform in a position similar to that of take-off.

### 2.3. Experimental Design

Two trials were performed for each of the jumping tests (SJ, CMJ and BJ), and this procedure was identical for the pre- and the post-intervention testing session. A 10 s rest within and a 90 s rest between jumping tests were set. The best trial was retained for analysis and the jumping height was calculated from the flight time [23].

Using the data from these tests, we calculated two muscle-strength indices, the elasticity index (EI),and the upper limbs coordination index (ULCI) [24]. The EI was calculated from the formula (100 × (CMJ − SJ)/SJ) and the ULCI from the formula (100 × (BJ − CMJ)/CMJ).

### 2.4. Statistical Analysis

Statistical analyses were performed using IBM SPSS v.20.0 (SPSS, Chicago, IL, USA). Descriptive statistics were calculated for all data. In addition, before the analysis, the Shapiro-Wilk test of the normality of distribution and Levene’s test for homogeneity of variance were calculated for all variables. The inter-session coefficient of variation (CV) was calculated by dividing the pre-post standard deviation (SD) by the pre-post mean for each jumping test (SJ, CMJ, and BJ) and for each participant and multiplying by 100 to obtain the percentage value. Intraclass correlation coefficient (ICC) and the mean value of CV for each jumping test were used to analyse inter-session reliability. The 95% Confidence Interval (CI) was also calculated. Student t-test examined differences between groups and between pre- and post-intervention. Effect size was calculated by Cohen’s d [25] for all variables between pre- and post-testing. The magnitude of the difference was considered small (0.2), moderate (0.5), or large (0.8). A mixed linear model univariate ANOVA was used to assess the interaction between competitive level (elite and amateur) and time (pre- and post-intervention) on jump variables. When a global difference over time (pre and post) was determined, Bonferroni post hoc analysis was used to identify where changes occurred. The magnitude of the differences among splits was examined using effect size eta squared (η^2^) and was evaluated as following: small (0.010 < η^2^ ≤ 0.059), moderate (0.059 < η^2^ ≤ 0.138) and large (η^2^ > 0.138). Statistical significance was set at *p* ≤ 0.05.

## 3. Results

The EG showed higher body mass (9.94%, *p* < 0.05) and stature (4.57%, *p* < 0.05) than the AG. There were no differences in body mass index (BMI, kg·m^−2^) between the groups. The mean at baseline and post-intervention and mean differences, as well as effect sizes for all variables for groups EG and AG are presented in Table 1.

The reliability assessment shows an excellent measure by the ICC in all jumping test [SJ Pre 0.92 (0.82–0.97); CMJ Pre 0.94 (0.87–0.98); BJ Pre 0.93 (0.84–0.97); SJ Post 0.94 (0.87–0.98); CMJ Post 0.97 (0.92–0.99); BJ Post 0.96 (0.90–0.98)]. The coefficients of variation for SJ, CMJ and BJ were 5.78%, 3.18%, and 3.86%, respectively.

No significant differences were noted between baseline and post-intervention in SJ (*p* = 0.965 and *p* = 0.655; ES = 0.01 and ES = 0.09), CMJ (*p* = 0.742 and *p* = 0.211; ES = 0.04 and ES = 0.10) in any group, EG and AG respectively. Although an increase in SJ was observed in both groups (EG = 0.04 cm; AG = 0.39 cm), there was a decrease in CMJ (EG = 0.15 cm; AG = 0.55 cm). However, BJ was significantly reduced following the 45 jumps on group EG (*p* = 0.043; ES = 0.40) while AG increased no significantly (*p* = 0.569; ES = 0.08).

No significant differences were apparent with EI (*p* = 0.989 and *p* = 0.242) and ULCI (*p* = 0.114 and *p* = 0.205) in any group. AG showed a decrease in EI with a medium effect size (ES = 0.50). EG presented a decrease in ULCI with a medium effect size (ES = 0.75) and AG an increase with a smaller effect (ES = 0.37).

At baseline, no differences were observed in jumping height in CMJ between EG and AG (28.84 ± 3.86 cm and 24.63 ± 5.20 cm for EG and AG, respectively) (*p* = 0.053), while there were differences in jumping height in SJ (27.20 ± 2.68 cm and 22.38 ± 4.02 cm for EG and AG, respectively) (*p* = 0.05) and BJ (32.97 ± 2.92 cm and 28.09 ± 4.73 cm for EG and AG, respectively) (*p* = 0.011). Post-intervention, no differences were observed in jumping height in BJ between groups (31.58 ± 3.98 cm and 28.45 ± 4.81 cm for EG and AG, respectively) (*p* = 0.122). However, in SJ and CMJ were observed statistically significant differences between groups (*p* = 0.020 for both comparisons). According to the indexes, EI and ULCI presented no statistical difference between groups at baseline (*p* = 0.114 and *p* = 0.961, respectively), while statistical difference was observed in ULCI post-intervention (*p* = 0.034, ES = 1.07).

**Figure 3:**
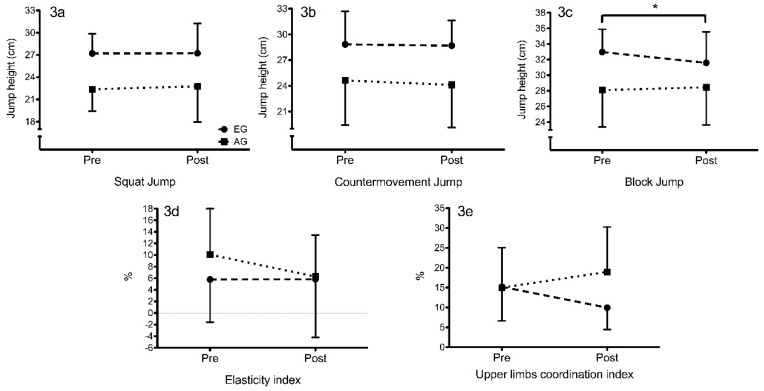
Comparison of jumps height between groups (mean ± SD). * = Significant effect (*p* ≤ 0.05).

A large group × intervention interaction on ULCI (*p* = 0.049, η^2^ = 0.189) was observed where ULCI decreased in elite and increased in amateur volleyball players in the post-test. Moreover, a large group × intervention interaction on BJ almost reached statistical significance (*p* = 0.064, η^2^ = 0.169) where the BJ likely decreased in elite and increased in amateur volleyball players (Figure 3).

## 4. Discussion

The purpose of the present study was to investigate the effects of a workout based on block jumps on jump height of female volleyball players with different level of competition (elite and amateur). The significant decrease in BJ jumping height for EG after the workout suggested that, although these players demonstrated the highest BJ height, their performance tended to decrease after multiple jumps. This decrease could be related with a possible fatigue induced by the intervention based on BJs [26], that consisted in the repetition of jumps with SSC [27]. This information is valuable to strength and conditioning coaches who are trying to maintain or improve the performance of their athletes during the competitive season. In most studies, the best players have higher VJ values [28,29]. Therefore, players from a national team have higher scores for spike and block jump differences in comparison to players from the university league [30].

The SJ showed non-significant increases (*p* = 0.965 and *p* = 0.655) in jump height of 0.2–1.7% in EG and AG, respectively, after the intervention. Although CMJ presented a decrease of 0.52–2.11% in jump height, this difference was not significant in any group, and the effect size was small. Previous studies reported increases in SJ and CMJ after an intervention. Nevertheless, the goal was to improve the jump performance, and the duration was between two weeks and a whole season [31,32,33,34]. Because SJ test measures “non-plyometric” displacement [35], results from the present study could be related with a possible muscle activation during the intervention, with acute positive effects on explosive jumping performance [36]. However, it takes a long training period to increase jump height in CMJ [37]. Therefore, it was expected to find a performance reduction post intervention in this test.

EG presented significant changes in BJ height with a decrease of 4.40% (*p* = 0.04; ES = 0.40), while AG had a non-significant increase of 1.27% after the intervention that was non-significant (*p* = 0.57; ES = 0.07). However, the main studies, which had measured BJ, were focused on changes throughout the season [5,31,38], and it is not possible to compare them with results observed in the present study. A decrease after intervention due to the possible fatigue was expected [39]. The increase in the blocking action produced after training periods could negatively affect AG subjects in comparison to EG subjects [40]. Fatigue could be a reason, since the ability to jump was reduced, according to previous studies [39,41]. Volleyball coaches and strength and conditioning coaches should focus attention on this factor in order to reduce injury risk [41,42,43,44].

EI is the percentage difference between SJ (explosive strength) and CMJ (elastic explosive properties) height [6,45]. EI showed different modifications after the intervention in both groups. EG presented a non-significant increase while AG had a non-significant decrease. Thus, after intervention, EG subjects maintain relatively (percentage) similar levels of elastic energy while AG experienced a statistically insignificant medium effect (ES = 0.50) decrease.

ULCI implicates a different percentage using arm swing for vertical jumping [24,46,47]. In contrast to previous index values, ULCI in EG showed a non-significant decrease with a medium effect size (ES = 0.75) and in AG a non-significant increase with a small effect size was showed (ES = 0.37). Therefore, after the intervention, EG showed less usage of their arms for vertical jumping in contrast with AG subjects. These results could explain the decrease in BJ jump height in EG subjects exposed previously. Both groups, in the last part of the season, performed the same exercise during the intervention, but EG had played in a more challenging league with a longer season than AG. This fact could therefore be the reason for EG’s decrease in performance after the intervention.

Arms have a significant role in generating jump height [46,48,49]. The performance when using an arm swing for jumping is related to the energy built up by the arms [47], so if fatigue after a high number of repetitions affects arms use, it could affect the jump height and consequently the BJ performance at the end of a game. Therefore, the appropriate use of the arms in BJ is a matter of discussion. In the present study, an intervention based on jumping was enough to produce significant performance decrease in BJ height in EG. However, there is no concern about arm swing leading to reduced performance, suggesting that it is well worth sacrificing jump height for the purpose of making a reactive movement faster and go directly to the block without a strong arm swing [37]. Future research should address which number of jumps is optimal for maintaining jump performance in defence during a game.

Comparing both groups, a difference was observed at baseline between them in SJ and BJ, as was expected, because jumping ability is a potential performance predictor in volleyball [29]. In addition, Sattler et al. [50] observed significant differences in VJ height between different play levels (most pronounced in the SJ). In the present study, differences in BJ between groups could be due to the starting position. Subjects started from a squat position (Squat BJ), which is related with explosive strength, and both groups presented initial differences in SJ height. However, no difference was observed between groups in CMJ at baseline [2,4,30,31,51,52]. Also, no difference was found in EI and ULCI at baseline. After the intervention, the statistical difference observed between groups in CMJ and ULCI was statistically significant (*p* = 0.020 and *p* = 0.034, respectively), and, in contrast, no statistical difference was shown on BJ. These results could be a consequence of a less use of arm swing by EG subjects, as it has been explained previously. Therefore, if a maintenance of arm swing is needed, training should include the ability to jump with minimal reduction in performance due to fatigue [53].

A limitation of the present study was the selected exercise protocol, which cannot accurately reproduce the fatigue conditions of a game. Although it consisted of a number of BJs similar to those performed during a game, it was not possible to simulate all the conditions of a real game (e.g., effect of opponents, random frequency of BJs, beginners vs. substitutes). In addition, the decrease of BJ height depended on rest performance, which might vary during a season [54]. The present study was conducted at the end of the competitive period where the jumping performance should be considered higher in comparison with the preparatory period or the beginning of the competitive period. Thus, it should be assumed that a lower initial BJ would be expected if the exercise protocol was applied in other training periods and, consequently, a smaller effect on BJ post-intervention would be observed. Another limitation is the difference in the number of training days between groups, which is a consequence of the level of the players.

## 5. Conclusions

The present study demonstrated that a number of BJs similar to those performed during a game resulted in decreased height of BJ in elite female volleyball players, which may affect their performance. In addition, the results of this study suggest that the number of jumps do not reduce explosive strength manifestation. Testing VJ performance during a training session and game could be useful to improve the knowledge about fatigue effects.

## Figures and Tables

**Table 1:** Characteristics of elite group (EG) and amateur group (AG).

Variable	EG (*n* = 10)	AG (*n* = 11)	Δ%	*p*	*d*
**Weight (kg)**	68.34 ± 7.97	61.55 ± 5.70	9.94	0.009	0.99
**Height (cm)**	175.70 ± 5.33	167.00 ± 7.87	4.57	0.036	1.32
**BMI (kg/m^2^)**	22.08 ± 1.88	22.07 ± 1.45	0.05	0.985	0.01

Δ%: differences in %; *d*: effect size.
